# Chimerical Dataset Creation Protocol Based on *Doddington Zoo*: A Biometric Application with Face, Eye, and ECG

**DOI:** 10.3390/s19132968

**Published:** 2019-07-05

**Authors:** Pedro Lopes Silva, Eduardo Luz, Gladston Moreira, Lauro Moraes, David Menotti

**Affiliations:** 1 Computing Department, Federal University of Ouro Preto, Ouro Preto 35400-000, MG, Brazil; 2 Department of Informatics, Federal University of Paraná, Curitiba 81531-990, PR, Brazil

**Keywords:** biometric systems, *Doddington Zoo*, multimodal biometrics, chimeric dataset, deep learning, deep representation, ECG, eye, face

## Abstract

Multimodal systems are a workaround to enhance the robustness and effectiveness of biometric systems. A proper multimodal dataset is of the utmost importance to build such systems. The literature presents some multimodal datasets, although, to the best of our knowledge, there are no previous studies combining face, iris/eye, and vital signals such as the Electrocardiogram (ECG). Moreover, there is no methodology to guide the construction and evaluation of a chimeric dataset. Taking that fact into account, we propose to create a chimeric dataset from three modalities in this work: ECG, eye, and face. Based on the *Doddington Zoo* criteria, we also propose a generic and systematic protocol imposing constraints for the creation of homogeneous chimeric individuals, which allow us to perform a fair and reproducible benchmark. Moreover, we have proposed a multimodal approach for these modalities based on state-of-the-art deep representations built by convolutional neural networks. We conduct the experiments in the open-world verification mode and on two different scenarios (intra-session and inter-session), using three modalities from two datasets: CYBHi (ECG) and FRGC (eye and face). Our multimodal approach achieves impressive decidability of 7.20 ± 0.18, yielding an almost perfect verification system (i.e., Equal Error Rate (EER) of 0.20% ± 0.06) on the intra-session scenario with unknown data. On the inter-session scenario, we achieve a decidability of 7.78 ± 0.78 and an EER of 0.06% ± 0.06. In summary, these figures represent a gain of over 28% in decidability and a reduction over 11% of the EER on the intra-session scenario for unknown data compared to the best-known unimodal approach. Besides, we achieve an improvement greater than 22% in decidability and an EER reduction over 6% in the inter-session scenario.

## 1. Introduction

Currently, robust and trustful mechanisms are required to protect our privacy, in particular when such information allows access to valuable goods or restricted places. In this direction, the digital methods for personal recognition are fundamental, and the most common practice is still using a Personal Identification Number (PIN) or merely a password.

Password-based approaches usually are related to something familiar to the subject/individual or it is written in somewhere and also encrypted, then digitally stored. This scenario is susceptible to several attacks in an attempt to steal important data. Due to these facts, more efficient ways to recognize an individual digitally have been investigated in the literature. In this context, biometrics-based techniques are the most promising path.

Biometrics approaches overcome such limitations encountered by PIN/password approaches because they use the human physiological or behavioral characteristics, which may have a high uniqueness factor and are more difficult to copycat/fake.

Governments and corporations finance several researchers, making biometrics a highly researched topic. The findings already have real usage, from simple smartphone access to terrorist identification in public spaces. Several biometric techniques have almost perfect accuracy in controlled environments [1], although this is not the case in unconstrained environments as in the mobile scenario [2] (also known as in the wild environments). Furthermore, individuals are often away from surveillance cameras, subject to different light sources, and may even be wearing accessories to deceive biometric systems purposely (wearing glasses, hats, make-up) in a real scenario. According to Neves et al. [3], there are no biometric systems capable of working with standard surveillance systems (a real scenario) currently. Thus, the unconstrained scenario is the most researchable one in the literature, and there are three significant patchworks to improve biometrics in these environments: improve anti-spoofing techniques, add robustness to digital modality representation, and by using multimodal systems. In this work, we focus on the last one.

A multimodal system is the one that uses two or more biometric modalities [4]. A biometric modality is a characteristic that can be used to identify or differentiate an individual. Some examples of biometric modalities are the face, the fingerprint, the iris, the voice, vital signals, and the gait. A great reason to use a multimodal system is that it is not possible to define which is the “best” biometric modality beforehand because each modality has a scenario where it works better. Moreover, from the ensemble classification theory, a diversity of sources can improve recognition rates. Based on this fact, each biometric modality can counterbalance the pros and cons of the others and may result in the enhancement of the performance.

In the literature, multimodal datasets are not so numerous as unimodal ones. Nonetheless, it is possible to find a few standard contemplating modalities (the most common), such as West Virginia University Biometrics (WVU) [5] with six modalities: fingerprint, face, iris, palm-print, and geometry from the hand and voice; Mobile biometrics (MOBIO) dataset [6] with face and voice; and Multimodal Database Captured with a Portable Handheld Device (MobBIO) [7] with face, eye, and voice, among others [8,9]. However, to the best of our knowledge, there are no datasets that mix the so-called standard modalities (face, eye, fingerprint) with novel and unusual biometrics, such as the ones provided by vital signals (for instance, from the heartbeat signal). We believe that biometrics from vital signs can contribute to the robustness of multimodal systems, especially for authentication in a mobile scenario [10]. Nonetheless, such investigation is not yet possible due to the lack of multimodal datasets. Taking this fact into account, our motivation for chimeric datasets arises.

A chimeric dataset is built by creating a set of chimeric individuals where each modality comes from a different dataset. More specifically, a chimeric individual is created by selecting the samples of a given individual from each kind of modality. The number of possible different combinations of the chimeric dataset (a set of chimeric individuals) is exponential to the number of subjects of each considered unimodal modality.

According to Wayman [11], the modalities of an individual are correlated with each other, and therefore, chimeric datasets are not the ideal condition. However, in the absence of multimodal datasets in the literature, chimeric datasets are the only option to investigate and understand the potential impacts of using unusual/new modalities together to enhance biometric systems.

It is worth mentioning that there is no standard protocol in the literature guiding the creation of chimeric datasets. It is still a point of discussion how to combine multiple biometric datasets. Thus, the present work addresses the chimeric datasets, creation for biometric purposes. The experiments handled in this work reinforce the use of multimodal systems for authentication in mobile devices, such as smartphones and tablets. Results show the advantages of multimodal systems, which in this work is a combination of three modalities described by deep representations: face, eye, and ECG. The most common manner to create a chimeric dataset is by randomly associating individuals among different datasets.

Usually, an approach based on random association does not take into account the nature of the different modalities or the specific degree of difficulty of each modality. This fact may cause one modality to stand out over others. This scenario may offer an optimist evaluation of an algorithm and be far from reality. Based on this fact, it is necessary to define the categorization of the individuals’ features. Based on this scenario, a classification scheme aiming to categorize the individuals according to their features was proposed in Doddington et al. [12]. Even though the categorization came from the work proposed in [12], our approach relies on the work proposed by Ross et al. [13]. Based on *Doddington Zoo*, the authors analyzed whether it was better or not to fuse two or more modalities. The authors also created several chimeric individuals to support their claims. Our approach uses the same concepts proposed in Ross et al. [13], aiming to add a constraint criterion to allow the creation of more homogeneous chimeric datasets. The objective of the present work is to promote a fair and controlled benchmark creation to compare methods.

We conduct several experiments addressing different scenarios aspiring to understand the effects of different types of fusion with a systematic and reproducible protocol. Furthermore, new periocular and face recognition models for verification in the open-gallery scenario are proposed, achieving a new State-Of-The-Art (SOTA) on the Face Recognition Grand Challenge (FRGC) dataset.

Therefore, we can summarize the contributions of this article as: (i) a fair and reproducible chimeric dataset creation protocol, (ii) investigation of the fusion of two traditional modalities (face and eye) with a biometric modality from the heart/off-the-person ECG, (iii) a more feasible and real application-prone method, and (iv) new SOTA results for the FRGC dataset on periocular recognition with verification mode.

This work is organized as follows. Section 2 shows works in the literature dealing with chimeric datasets. A review of the considered datasets and the state-of-the-art methods is described in Section 3. Section 4 presents the proposed methodology to create the chimeric dataset. The experiments are presented along with the results and discussion in Section 6. Finally, conclusions are highlighted in Section 7.

## 2. Related Chimeric Datasets’ Approaches

Due to the lack of public datasets and benchmarks at the moment, evaluating multimodal systems is a challenge. Besides, there are no datasets that consider all possible biometrics’ combinations. However, if one wants to study an unusual combination of modalities, there are a couple of alternatives. One is the creation of a new dataset, which is expensive and often a difficult task. Another option is to create a chimeric dataset. In this section, we describe a few works related to chimeric datasets regardless of biometric usage.

In [14], a chimeric dataset with two modalities was proposed: fingerprint and ECG. The ECG signal was used both for liveness and recognition, although the focus of that work was liveness detection. One significant contribution relied on the stop criterion proposed to reduce the size of the sample signal. The authors reported an EER of 3% for liveness detection with thirty seconds of the acquired signal. The chimeric dataset was randomly built, pairing one subject from the ECG dataset with another subject in the fingerprint dataset. The ECG dataset was collected by the authors in BioSec lab at the University of Toronto, while the fingerprint came from the LivDet2015 dataset [15]. Five hundred different subjects were created, and the process was repeated five times due to the randomness of the dataset construction.

Another chimeric approach involving the on-the-person ECG signal was the one proposed by Singh et al. [16], which also employed fingerprint and face for the recognition task. The ECG data came from the Physionet dataset, while the fingerprint and face data came from NIST. The authors investigated a fusion in a score level scenario, focusing on the weighted sum rule. The reported EER, for the unimodal scenario, was 10.80% for the ECG, 4.52% for the face, and 2.12% for the fingerprint. After fusion, the EER significantly dropped to 0.22%.

Barra et al. [17] proposed a new chimeric dataset by merging the ECG signal with six bands of EEG signals. Their recognition approach rested on fiducial points of the ECG signal along with features extracted from the EEG spectrum. Five random non-overlapped segments from a total of 12 s of each signal were employed, in which one segment was randomly chosen for the probe set, while the remaining were used as a gallery. One individual from the ECG dataset was arbitrarily combined with another individual from the EEG dataset, resulting 52 chimeric individuals. The authors also explored different EEG channels. The fusion was performed in a score level scenario with Euclidean distance as the metric distance. Three fusion rules were investigated: the sum, the product, and the weighted sum. The best result, reported on five-fold cross-validation, was the one considering the weight sum and EEG delta band, achieving an EER of 0.928%.

One behavioral biometry, very common in the literature, is through the handwritten signature. In [18] was proposed an approach for feature-level fusion applying the signature and face. Wavelet-based features were used to represent both modalities. The chimeric dataset was created by randomly associating a face with a signature, which resulted in 30 chimeric individuals. A Hamming distance classifier was employed for the genuine or impostor decision. The reported accuracy was of 97.5% for the Olivetti Research Ltd (ORL) dataset (face) plus the Caltech dataset (signature) and 98.88% for the ORL Ucoer dataset (signature). According to the authors, the fusion of the modalities delivered better effectiveness than considering the modalities alone.

Two of the most promising modalities in the biometric scenario, the iris and the face, were merged in [19] on a score level fusion using a weighted sum rule. The weights were empirically chosen and were specific for each subset of data. The authors reported an accuracy of 99.4% and used three well-established datasets in the literature: Universiti Teknologi Malaysia Iris and Face Multimodal Datasets (UTMIFM), UBIRIS Version 2.0 (UBIRIS v.2), and ORL face. The UTMIFM is a dataset with both modalities and the chimeric dataset built on UBIRIS v.2 and ORL stochastically.

Due to the different nature of chimeric datasets, in the literature, there is no standard protocol to collaborate with the creation of such datasets. Furthermore, the reported process of creating the chimeric dataset was unclear for the methods cited above and had a lack of details, and most of the authors did not perform a proper statistical test.

In this work, we propose a comprehensible and reproducible protocol for the creation and evaluation of a chimeric dataset in Section 4.

## 3. Biometric Modalities’ Review

In this section, two datasets considered in this work (CYBHi and FRGC) are explained in detail, as well as the SOTA techniques for each modality are described.

### 3.1. Check Your Biosignals Here Initiative (ECG)

**Figure 1:**
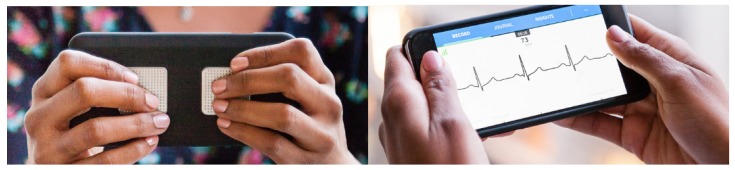
Example of the commercial off-the person mobile equipment used to capture the ECG signal. Source: https://www.alivecor.com/en/.

The Check Your Biosignals Here initiative (CYBHi) [20] is an off-the-person ECG dataset, which means that the data were acquired without the use of specialized electrodes on the chest or conductive gel. The data acquisition was performed only by finger contact, and it can be considered an unconstrained dataset, since it is less invasive and offers more challenges for the acquisition process. In our opinion, investigating the combination of heart signals as biometrics, especially the off-the-person ECG, with other standard biometric modalities is a promising research path, since its real usage can be made possible with portable and wearable devices, such as the one presented in Figure 1. Furthermore, some researchers are studying how to capture heart signals through a video camera, which may be even more interesting for biometrics derived from the heart [21,22].

**Figure 2:**
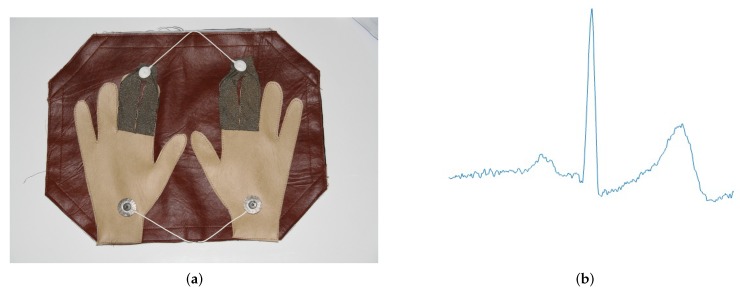
Example of the device and the ECG from the CYBHi dataset. (**a**) Example of the device used to get the ECG signal. Source: [20]. (**b**) Example of the ECG shape extracted from the CYBHi dataset.

The technique used on the CYBHi dataset acquired the data by the fingers and hand palms, using a proper device, as the prototype shown in Figure 2a. Figure 2b is an example of the resultant ECG extracted with the off-the-person ECG device.

The dataset was acquired in two setups: one called short-term signals and the other long-term signals. In this work, the long-term signal setup was used. The data acquisition occurred in two different moments, also called sessions, separated by a period of three months. The rationale of these experiments was to study the ECG morphology over time. In both sessions, the ECG signal was acquired from the fingers, and for both sessions, the subject remained seated during two minutes in a resting position with a sample rate of 1 kHz.

The authors used a total of 63 subjects; most of them were nursing or health technologies students. All 63 subjects participated in the two sessions. There were 49 females (78%) and 14 males (22%), with an average age of 20.68 ± 2.83. None of the samples reported any health problems, which represents the average population.

#### SOTA

In [10], an approach based on deep learning, specifically convolutional networks, was proposed for the task of biometrics with the ECG signal. A short recall of this neural network is presented in Section 5.2.1. The features of the ECG signal were obtained from both the raw signal and a 2D spectrogram representation of the signal. The authors introduced a data augmentation technique, which improved the use of deep convolution networks. According to the authors, the proposed approach achieved SOTA results in two off-the-person publicly-available datasets (CYBHi [20] and UofTDB [23]).

### 3.2. Face Recognition Grand Challenge (Eye and Face)

**Figure 3:**
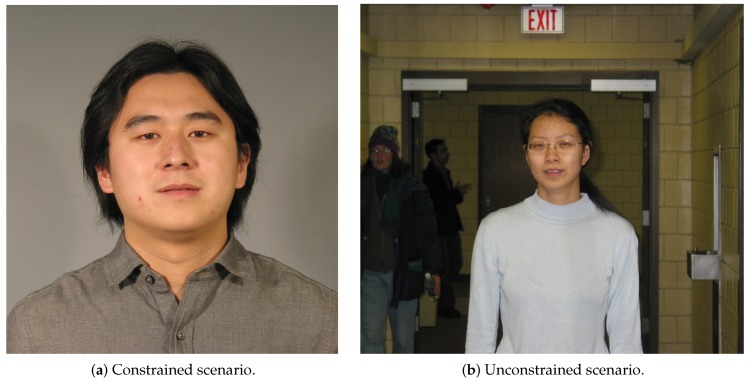
Example of FRGC face images. Source: [24].

The Face Recognition Grand Challenge [24], or just FRGC, is a dataset created to provide a progressively difficult challenge for face recognition. This dataset has 50,000 recordings, which are divided into training and validation partitions. The FRGC has different sets of data, from high resolution still images in constrained to unconstrained scenarios and 3D images. The constrained images were acquired in two different lighting conditions and two expressions (smiling and neutral) in a controlled scenario (in a studio with full frontal facial images). Similar to the constrained scenario, in the unconstrained images, the faces were captured with two facial expressions, although in different places, such as hallways, atriums, and outdoor. Since it is a hard task to create a 3D face model, the 3D images were taken under a controlled illumination scenario. Figure 3 shows some examples of the FRGC dataset.

As images have high resolution, it is possible to crop eyes from the face and still have enough information for biometric recognition. Based on that, a few works focused on in-the-wild (unconstrained) eye recognition research on FRGC datasets.

#### 3.2.1. SOTA for Eye Recognition

The current SOTA on eye recognition for the FRGC is the work proposed by Proença and Neves [25], in which a deep CNN trained with ocular information was proposed. The authors forced a CNN to disregard information from the iris and sclera, which improved the results for periocular biometrics in visible light. Furthermore, the preprocessing applied to images during training provided an efficient data augmentation technique. However, the work was focused on identification mode, i.e., closed world or closed gallery approach. For verification mode, the SOTA method was the one proposed in [26]. Although this work did not make use of deep learning representations, the method was robust and achieved SOTA results both in FRGC and UBIRIS.V2. The work in [26] proposed a recognition based on two sorts of biometrics, one called by the authors as strong (iris) and another called weak biometric (periocular region). The weak biometric was used as a complement for the stronger one. The iris features were extracted from the texture and the multispectral information in visible light. The features were extracted from a normalized iris (Daugman’s rubber sheet model) convoluted with a bank of Multi-Lobe Differential Filters (MLDF). For the periocular region, the authors focused on the shape of eyelids and the region among the eyelids, eyelashes, and skin wrinkles/furrows. These parts of the periocular region were used to set the boundaries of the region of interest. The author created an ensemble to fuse both strong and weak biometrics.

#### 3.2.2. SOTA for Face Recognition

The authors in [27] showed how the process of fine-tuning a deep convolutional network does not necessarily improve results when training and testing datasets come from different distributions. When there are significant differences among datasets, the performance of fine-tuning a pre-trained CNN is compromised. The proposed solution was to apply non-CNN features to the recognition process. Besides the CNN model, the authors also applied Principal Component Analysis (PCA) over the raw pixels of face images. The features from the CNN and the result from the PCA were both combined by the Mahalanobis distance to perform face verification. The authors reported a verification rate of 92.50% and 96.00%, using the CNN model alone and by adding the non-CNN features, respectively.

## 4. Multimodal Chimeric Dataset Creation and Evaluation Protocol

**Figure 4:**
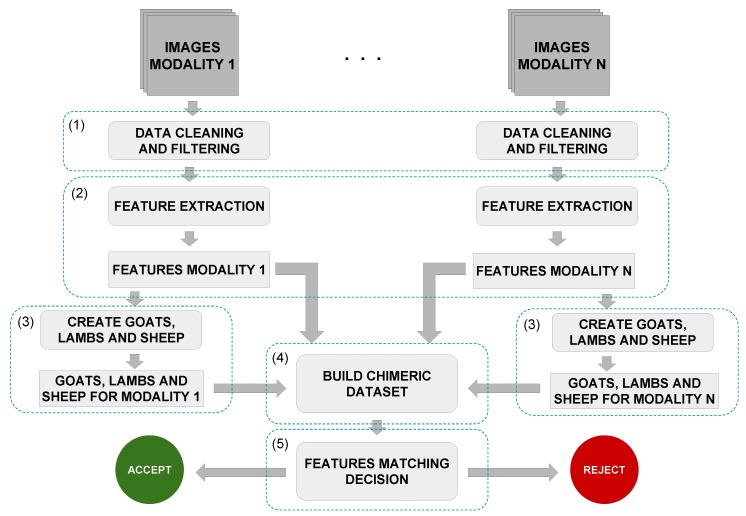
Recognition process of a chimeric individual.

In this section, the methodology to create a chimeric dataset from multiple datasets is presented. We also recommend how to evaluate and make a fair comparison of methods using a chimeric dataset. The overview of the methodology is shown in Figure 4. The process can be split into five steps: (1) cleaning and filtering the data (preprocessing), (2) feature extraction, (3) *Doddington Zoo* criterion, (4) chimeric individual creation and modalities fusion, and (5) feature matching and decision. The first three steps were executed independently for each modality, while the latter two were responsible for the fusion and the subject recognition.

### 4.1. Cleaning and Filtering Data

This step was executed over all unimodal datasets individually, as illustrated in Figure 4. This step included cleaning (or filtering), segmentation, and data normalization towards preparing the dataset for the next steps to facilitate the learning and feature representation processes. For instance, it may be used to identify and remove outliers, excessive noise, or any incorrect and corrupted data segment. It is worthwhile noticing that each biometric modality had its own set of methods and approaches to data cleaning since each one had its singularities. Furthermore, this step was not mandatory.

### 4.2. Feature Extraction

This step aimed to map a set of raw (or preprocessed) data to a more discriminating representation. This step is crucial in a machine learning context, and from that forward, each instance of one modality was represented by a feature vector. A feature vector typically carries relevant and non-redundant information in a compact view. The features must favor the learning and generalization process of training machine learning models and also facilitate the classification task.

### 4.3. Doddington Zoo Criterion

In a recognition system, there are always individuals who are more likely to be confused with others and those individuals with little intra-class variability, therefore being easily identifiable. The former individuals contribute more to false acceptance and false rejection errors and thus can distort results, especially when using EER to analyze the system performance [12,13].

In [12], an animal-based nomenclature was proposed to classify individuals into a recognition system, called the *Doddington Zoo*. According to Doddington et al. [12], most common or default individuals who are predominant in the population are labeled as *sheep*. Individuals that hard to recognize correctly, and therefore with larger intra-class variability, are called *goats*. Those individuals who are easily imitated and have lower inter-class variability are classified as *lambs*. Finally, those individuals who are easy to be confused with others or have potential to impersonate others are called *wolves*. Since the spoofing scenario was not explored here, we did not consider the wolf category for the present work.

In [13], the authors explored the use of the *Doddington Zoo* nomenclature to assist in decision making for merging two biometric modalities. These authors investigated several scenarios and concluded that fusion was the best option when two modalities to be fused came from two individuals labeled as *goat* or from two individuals labeled as *lamb*. According to [13], other combinations of individuals may favor one modality over another.

In this work, for each round of evaluation, a new (random) chimeric dataset was created, and therefore, if one does not control the combination of individuals using a criterion such as the *Doddington Zoo*, one could create a dataset on which a specific modality has a disproportional weight in the result, or it is possible to create a dataset with an excess of weak individuals (*goat* or *lamb* type). To avoid this situation, we used the criterion proposed by Doddington et al. [12] to label the individuals of the unimodal datasets, and we propose the following constraints for the combination:

1. A chimeric individual can only be created by combining individuals with the same label. Thus, the modalities that compose a chimeric individual must have the same level of difficulty, that is the same amount of each category of animals’ overall modalities. We considered that in datasets containing large numbers of *lambs* and *goats*, it tends to be harder to conduct a verification process, therefore more difficult;
2. The chimeric dataset must have a fixed number of chimeric individuals of the three labels: *sheep*, *goat*, and *lamb*;
3. The number of *sheep*-type individuals should be the majority case.

With these restrictions, we intended to favor the creation of more homogeneous chimeric datasets to promote reproducibility and comparison. Furthermore, the number of individuals categorized as *goat* or *lamb* was much lower than the number of individuals labeled as *sheep* from the unimodal point of view. Other combination restrictions were explored in [13] for two modalities. We selected the combinations that favored the fusion of modalities and reduced the impact of a single modality standing out from the others.

To determine the category of the individuals, we used the methodology proposed in [13] and the training data. First, all individuals without at least one sample in any session were discarded. To determine who must be labeled as *goat*, a few steps should be taken. First, the intra-class distance was calculated for all users and sorted in an ascending manner. After that, the last *n* individuals were selected, which represented all individuals above the 70th percentile. The rationale for this was to select individuals with the largest distances from the intra-class samples. For the *lambs*, first, the mean inter-class distance was calculated in a one-against-all comparison. Then, results were sorted in ascending order. Thereafter, the first *n* individuals who represented the lower 30th percentile were selected. The rationale of this approach was to select individuals with a lower distance difference from others. Equation (1) was used for percentiles’ calculation. This equation returns the index related to one specific percentile, and thus, the individual above or lower this index is considered a *lamb*. It is worth highlighting that the individuals here were described as integers and each individual was labeled as only one animal.

(1) $$index=\frac{p}{100}\times N+\frac{1}{2}$$

in which *p* is the percentile and *N* is the number of individuals available in the dataset.

We have two possible scenarios: (1) modalities that come from different individuals and (2) modalities that come from the same individual. For the first scenario, the process was conducted separately for each modality since there was not a strong correlation among individuals. For the second scenario where modalities belonged to the same dataset, we forced the chimeric individual to share the same label among all modalities. To define the category (*goats*/*lambs*) of those chimeric individuals, we first detected categories separately in each modality and chose those that shared the same category. However, this approach may result in a total of individuals lower than the 10th percentile expected. To circumvent this problem, the following criteria were used: (1) individuals who had divergent labels, and therefore were excluded from the first round of chimeric individual composition, were now considered; (2) a new random permutation was created from this new set; (3) the first nindividuals were then used to complete the category.

Once the *goats* and *lambs* were defined, we generated a random permutation of them, selected a proportional 10^th^ percentile of valid individuals of each “animal”, and set the remaining as *sheep* individuals. With that, we aimed to generate different chimeric datasets in each execution.

Once the *goats*, *lambs*, and *sheep* were determined for each modality, the *goats* were combined with other *goats*, *lambs* with *lambs*, and *sheep* with *sheep*. The amount of each “animal” was limited according to the modality with the lowest number of an animal available. For instance, if Modality 1 had 10 *goats*, 10 *lambs*, and 80 *sheep* and Modality 2 had 5 *goats*, 5 *lambs*, and 400 *sheep*, the chimeric dataset created over these two modalities would have 5 *goats*, 5 *lambs*, and 80 *sheep*.

### 4.4. Building the Chimerical Dataset

The first two steps were performed only once. For each modality, a specific feature extraction method was applied and the feature vectors stored. Those feature vectors were used to label individuals from unimodal datasets according to the *Doddington Zoo* criteria. In that sense, the chimeric individuals were created with pre-computed features. The proposed chimeric dataset creation was a guided stochastic process because of the *Doddington Zoo* criteria, and thus, for each new experiment, a new chimeric dataset had to be created. To promote reproducibility, we stress that the method and the seeds used to generate randomness should be stored and made available.

The process of creating the chimeric dataset can be formally expressed as follows. Let *D* be a dataset scenario with different modalities, with 1≤i≤m, i.e., $D=\{M^{1},M^{2},\ldots ,M^{m}\}$. Furthermore, let $M^{i}=\left\{I_{j,k}^{i}\right\}$ be the set of individual samples of the modality *i*, where $I_{j,k}^{i}$ is the $k^{\mathrm{th}}$ sample of the individual *j* of the modality $M^{i}$. Note that each modality may have a distinct number of individuals and each individual a different number of samples. Then, let n(i) be the number of individuals for the modality *i* and s(j) the number of samples for each individual *j* of modality *i*, with j∈{1,…,n(i)} and k∈{1,…,s(j)}.

Then, a chimeric individual (or subject), denoted by $I^{C}$, is defined as follows, $I^{C}=\left(I_{j_{1},k_{1}}^{1},I_{j_{2},k_{2}}^{2},I_{j_{3},k_{3}}^{3},\ldots ,I_{j_{m},k_{m}}^{m}\right)$ for some $j_{i}\in \left\{1,\ldots ,n\left(i\right)\right\}$ and $k_{i}\in \left\{1,\ldots ,s\left(j\right)\right\}$, chosen only once. Hence, the number of individuals of the chimeric dataset was equal to the number of individuals of the modality with fewer individuals. Besides, the number of samples of each chimeric individual was limited by the lowest number of samples of the selected individuals of each modality. The number of samples may vary for each chimeric individual. We stress that this process was accomplished separately for each category (label) regarding the *Doddington Zoo* criteria and respecting the restrictions presented in Section 4.3.

**Figure 5:**
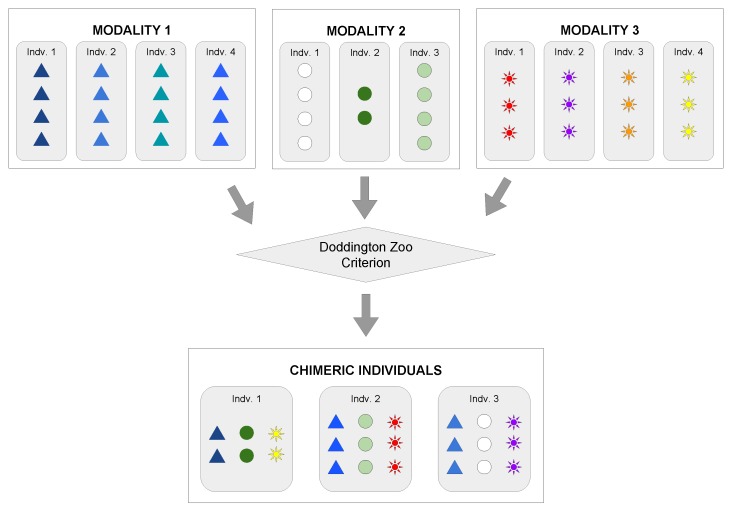
The process to combine modalities and create the chimeric individual is a stochastic process, respecting the constraints presented in Section 4.3. The figure illustrates the process of creating chimeric individuals of one specific *Doddington Zoo* category.

To understand the creation process of a chimeric individual, one can consider a scenario with three datasets and each dataset representing a different modality. From this scenario with three modalities, one individual was randomly selected from each dataset and combined with another two individuals from the other two datasets, respecting the label and restrictions presented in Section 4.3, thus resulting in a new chimeric individual. The samples of each modality were combined sequentially, that is the first sample of Modality 1 was combined with the first sample of Modality 2, Modality 1’s second sample was combined with Modality 2’s second sample, and so on. All data belonging to this new chimeric individual were then excluded from the next rounds of selection, ensuring that a single individual from a dataset A was assigned only to a single individual from a dataset B, and the process was repeated until there were no more available individuals for selections on the datasets. As this association was made one by one (one individual of Modality 1 was combined with one of Modality 2 and one of Modality 3, as illustrated in Figure 5), the amount of individuals was limited to the dataset with the lowest number of individuals, and the number of samples per chimeric individual was also limited by the most restrictive modality of one specific individual.

Figure 5 illustrates the proposed chimeric dataset protocol with a didactic example, in which the amount of individuals on Modality 2 dataset was the one that limited the total number of chimeric individuals to be generated and the number of samples of Modality 3 limited the number of total samples per chimeric individual. The second individual of Modality 2 also limited the number of samples of one chimeric individual.

### 4.5. Feature Matching and Decision

Once the chimeric dataset was created, the data could be used to perform a biometric recognition and, the next natural step, to evaluate and compare the methods as well. In biometric systems, a common evaluation mode is called verification or positive identification, where the goal is to prevent more than one person from accessing the same information [28]. The *verification mode* is a challenging evaluation since it resembles the open world/gallery case. Thus, our protocol recommended the use of verification to compare works.

In the *verification mode*, the biometric system validated whether a subject was whom he/she claimed to be. That is, the input information was compared against the identity data previously informed to the system. Verification is widely used, and the most common forms are alphanumeric passwords and token cards. Considering verification by similarity, a verification system can be mathematically modeled as in Equation (2):

(2) $$\left(I,X_{q}\right)\in \left\{\begin{array}{cc} w_{1}, & S(X_{q},X_{I})>t\\ w_{2}, & false \end{array}\right.$$

where *I* is the identity to be verified (an integer number denoting an individual label), $X_{q}$ is the input feature vector, $w_{1}$ is the genuine subject class, $w_{2}$ is the impostor subject class, *S* is a function that measures the similarity between two feature vectors (with the same dimensionality): $X_{q}$ and $X_{I}$ (comparison between two instance pairs), and *t* a pre-defined threshold [28]. The similarity (*S*) was calculated using some metric distance. The most common distance metrics are the Euclidean distance, Manhattan distance, Mahalanobis, Spearman distance, and Hamming distance.

It is important to note that the verification modes used a threshold of *t* in the comparison. Two samples from the same subject, even on the same sensor, may present variations between them. These variations can be generated by the subject himself/herself (for example, a different haircut or a different pose) or even by sensor imperfection (noises). The threshold used directly affects the system effectiveness, and finding the most appropriate one depends on the application. It is common in the literature to construct distribution curves (histogram of scores), as well as the Detection Error Trade-off (DET) curve for method evaluation. From the DET curve and the score distribution curves, two different metrics could be used to evaluate the methodology: decidability and Equal Error Rate (EER). Both are widely used in the literature to compare methods on several publicly-available datasets.

The decidability (*d*) indicates how far the intra-class (genuine) distribution scores are from the inter-class (impostor) ones [29]. The calculation relies on the means and standard deviations of the intra-class ($\mu_{I}$ and $\sigma_{I}$) and inter-class distribution ($\mu_{E}$ and $\sigma_{E}$), and it can be expressed as:

(3) $$d=\frac{\left|\mu_{E}-\mu_{I}\right|}{\sqrt{\frac{1}{2}\left(\sigma_{I}^{2}+\sigma_{E}^{2}\right)}}.$$

The EER is related to the False Acceptance Rate (FAR) and the False Rejection Rate (FRR). The EER is the point where the FAR is equal to FRR. The lower is the EER, the better is the system in the average case. The EER can be calculated from the DET curve, which plots the false rejection rate *vs.* the false acceptance rate, as being the point that crosses the curve or the closest point to the perfect one.

When working with multimodal datasets, such as the ones used on the proposed chimeric dataset, a criterion must be applied to merge the modalities. However, how to combine/fusion modalities properly is still an open problem in the literature, and it may happen at four different levels: sensor, features, scores, and decision. The fusion at the sensor level consists of merging two or more input data before any recognition process, for instance fusion images in the infrared with the ones in the visible spectrum to perform face recognition. The fusion at the feature level is conducted after the feature extraction step. The merge may occur in several manners with the simplest one being the simple concatenation: *n* features of one modality concatenated with *m* features of another modality, which results in a new set of features with an n+m vector size. This scenario is the one with the biggest potential for optimization in multimodal systems; once there is more information available.

The fusion at the score level also happened after the feature extraction step. The similarity scores were computed separately for each modality and combined employing a specific rule. The most simple and common rules used to combine scores are the sum, product, max, min, and exponential sum. It is also possible to use more complex strategies. The fusion at the decision level is less popular in the literature and is often associated with classifiers and the voting strategy.

### 4.6. Comparing Results

Due to the randomness of the chimeric dataset creation process, one must consider the law of large numbers. In that sense, for a fair comparison, we recommend executing the entire random process 30 times [30]. Thus, one must compute the mean and standard deviation and perform a statistical test to compare the methods.

Besides, one should pay attention to whether datasets have different acquisition sessions. Two scenarios should emerge: the intra-session and the inter-session. In this context, the intra-session scenario means all the images are taken in a short period of time, usually in the same place and at the same moment. The inter-session scenario means all the images are taken at larger time intervals (days or even weeks apart). The inter-session scenario produces a “natural noise” due to its own individual interference, such as changes in haircut, skin tone, accessories, and makeup, among other changes. The inter-session problem tends to be harder than the intra-session problem since, for the inter-session problems, the variation among samples from the same class (same individual) is higher when compared to the intra-session problem. Since the proposed protocol aimed to evaluate the robustness of the data representation (features), we recommend evaluating methods in at least two sessions, to assess the impact of intra-class variation in time.

To perform the evaluation, one score should be computed for each pair of samples of the dataset, in a one-against-all fashion. For the intra-session scenario, only one set of data (one session) is considered for both training and testing. However, for the inter-session scenario, data from two or more sessions should be used. Thus, all the pairs are necessarily created with data from different sessions.

## 5. FEyG Dataset Creation

In this section, we employ the proposed methodology for chimeric dataset creation described in Section 4, by describing the specific data cleaning (preprocessing), feature extraction, feature matching, and decision processes for the datasets considered here. We name the chimeric dataset, created by exploring the three modalities previously described in this work, as FEyG (**F**ace, **Ey**e, and EC**G**). Furthermore, the details of the employed techniques are also described.

### 5.1. Cleaning and Filtering the Data

Different approaches were applied for each modality since each one had its specificities. We emphasize that this process was not mandatory for all modalities or datasets.

#### 5.1.1. ECG

The same preprocessing approach proposed in [10] was adopted here. The preprocessing was distributed into two steps: heartbeat segmentation and outlier removal. The heartbeat segmentation involved the use of a forth-order, zero-phase Butterworth bandpass filter with cutoff frequencies at 0.5 Hz and 40 Hz. After the filtering step, the QRS complex was detected by the Pan and Tompkins algorithm [31], and the heartbeat was segmented with an 800-ms window centered at the R peak. Next, outlier removal was applied to exclude highly-corrupted heartbeats or segmentation mistakes, by disposing of 15% of the heartbeats in which the R peaks were distant from the mean heartbeat. After the filtering and outlier removal process, the 4259 remaining heartbeats belonging to 56 individuals composed our ECG dataset.

#### 5.1.2. Face and Eye

For the FRGC dataset, all images were considered, regardless of whether they were unconstrained (Figure 3a) or constrained (Figure 3b). However, only images where the face was detected were considered. A facial landmark detector algorithm was applied to detect the face (library available at https://pypi.org/project/dlib/), which was created by training an ensemble of regression trees on the Intelligent Behaviour Understanding Group (iBUG) 300-W dataset [32]. The detector found coordinates of landmarks corresponding to the regions that surround each facial structure, such as the mouth, nose, eyebrows, chin, and eyes. The landmarks were estimated without feature extraction and directly from the pixels [33]. The method returned 68 points for each face detected. Based on those 68 points, both eyes were cropped and the face itself segmented.

**Figure 6:**
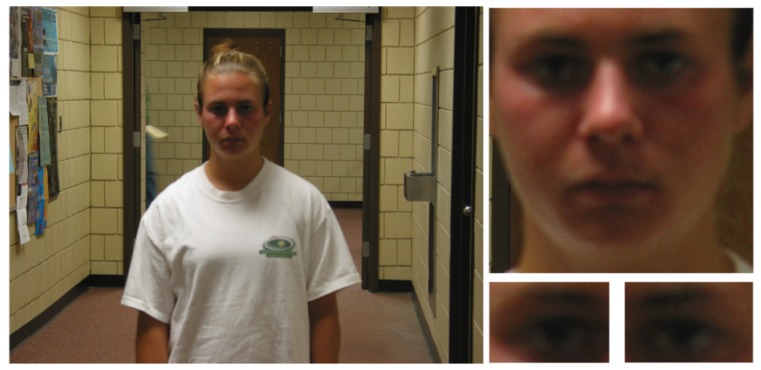
Example of the crops made from a random image of the FRGC dataset. Source: [24].

When two or more faces (Figure 3b) were detected in the same image, only the largest one was chosen. When no face was detected, the image was discarded. Such a methodology resulted in 32,794 images from 314 individuals. An example of such a segmentation can be seen in Figure 6.

### 5.2. Feature Extraction

Deep learning techniques are used to represent the images/signals of the modalities, since it represents the SOTA for all datasets considered here. The method proposed in [10] was reproduced to extract the deep representations for the ECG signal. For face and eye, two new models were created here, in which the two deep models were fine-tuned using transfer learning from pre-trained VGG models (see the SOTA techniques described in Section 3). For reproducibility purposes, the source code for all steps will be made publicly available once this article is accepted for publication (http://www.decom.ufop.br/csilab/).

The methodology for feature extraction consisted of proposing convolutional network architectures, based on the literature and the specific problem, and training them, with the aid of data augmentation or transfer learning, for the simple classification task. Once trained, the network was then no longer used for classification, but data representation. Once the features were extracted and stored, they were used for evaluation in the biometric verification mode (open world problem).

#### 5.2.1. ECG

For ECG data representation, we considered the best architecture presented in [10], and we used the same technique for data augmentation.

**Figure 7:**
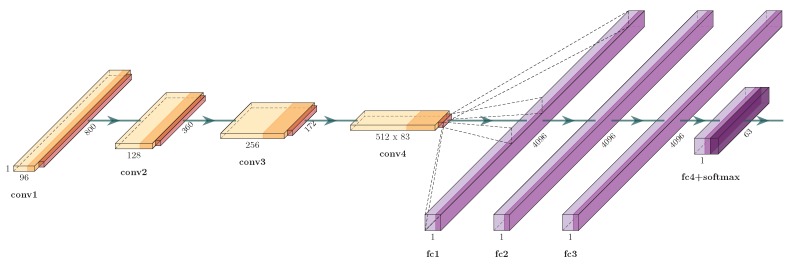
CNN proposed in [10] aiming at ECG recognition for the verification task.TIFS ECG

The convolutional neural network architecture (See Figure 7) was feed-forwarded with an 800-ms window input that represented the ECG signal. Following [10], the CNN architecture started with a convolutional layer with 96 filters and a stride equal to one with padding to preserve the original size of the signal. The next layer was a max-pooling layer aiming at a subsample of the signal (stride>1). Using the same stride and padding, three convolutions and max-pooling were used, with 128, 256, and 512 filters in sequence. The last layers were *Fully-Connected* (FC) layers, dropout (10%) and softmax-loss, where three FC with 4096 neurons in a row and the last one with 63 neurons (number of classes) with softmax.

After the training process, the last layers (the softmax, dropout, and FC related to the class prediction) were removed, and in this manner, the CNN became a feature descriptor model. For training, stochastic gradient descent with mini-batches of size 100 and momentum of 0.9 was considered. A learning rate of 0.01 was used for the first 40 epochs, then 0.001 for more 20 epochs, and finally, 0.0001 for the remaining. The weights of filters were randomly initialized with a zero mean Gaussian distribution and deviation of 10^−2^. During the training phase, 10% of the data was reserved for validation.

#### 5.2.2. Eye

**Figure 8:**
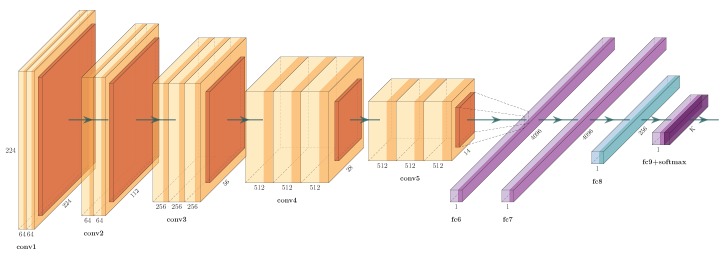
Periocular Region Recognition (PRR)-256 proposed in [2] aiming at periocular recognition for the verification task.VGG

In order to create a deep representation, the VGG model trained for eye representation, proposed in [2] (PRR-256 in Figure 8), was used as a starting point for the transfer learning [34] process employed here.

Following the training methodology proposed in Luz et al. [2], all layers of the PRR-256 network, except the last one, were transferred, and the network was trained as if it were a common classification problem (closed gallery, fixed number of classes). The last one was customized to handle the specific number of classes of the target dataset, here the filtered dataset after the preprocessing described in Section 5.1.2. Thereafter, the PRR-256 was fine-tuned with images from both eyes for 30 epochs (first epoch with a learning rate of 0.001, ten with 0.0005, ten with 0.0001, and finally, the last ten epochs with 0.00001). In total, 23,148 images were used to train (11,574 for each eye) and 2572 images for validation (1286 for each eye). After training, the last two layers of the network were removed, and the model was used as a data representation model, in which, for each image that was presented as the input of the network, a feature vector of 256 dimensions was produced. We used the fall season images as the training data and the spring images’ data as the test data.

From the test set of 39,868 images with no overlap with the training images’ set (19,934 for each eye), the protocol proposed by Proença [26] was carried out to compare with the SOTA method. From the test images, a fixed set of 50,000 intra-pairs and 250,000 inter-pairs was created and randomly selected. The experiment was repeated 30 times. The distance metric applied to achieve the matching score was the Spearman distance. Table 1 shows our approach against Proença’s approach (SOTA for the verification task).

Considering the results presented in Table 1, a new SOTA was achieved for periocular recognition in verification mode.

#### 5.2.3. Face

For the face, a deep transfer model, also based on the VGG, was trained. The VGG model is competitive, regarding other deep learning models, for face recognition tasks on challenging datasets, such as the Labelled Faces in the Wild (LFW) dataset [35] and the YouTube dataset [36], and therefore the considered SOTA method in this work. Furthermore, its pre-trained model is widely available. The FRGC dataset is also a challenging dataset and holds similarities to datasets in which the VGG model achieves SOTA results.

Again, a process similar to that proposed in [2] was followed, where all layers from VGG were transferred, except for the last one, to accommodate the number of FRGC individuals (dataset classes). The training and test data distribution followed the same one as that used with eye recognition. A learning rate of 0.001 was used to fine-tune the model at the first epoch and 0.0005 for the nine epochs remaining.

Once the whole model was trained, the last two layers (softmax and fully-connected with the number of the classes which is 314 individuals) were then removed. In that sense, the new last layer was the layer from which the features are collected.

It is essential to highlight that, before applying the fine-tuning on a VGG model, the whole FRGC dataset was preprocessed. Images whose face could not be detected by the segmentation algorithm were ignored.

## 6. Multimodal Chimeric Dataset Evaluation

In this section, we describe the experiments performed on the FEyG dataset. The analysis was performed in three fusion scenarios: a fusion of two and three modalities combined. We also present the case in which the modalities were evaluated separately, in order to assess the impact of the fusion regarding each different modality.

We conducted the experiments on an Intel (R) Core i7-5820K CPU @ 3.30 GHz with 12 cores, 64 GB of DDR4 RAM, and a GeForce GTX TITAN X GPU. All the implementations were based on the MatConvNet toolbox [37] linked with NVIDIA CuDNN.

The datasets considered here did not have the same amount of individuals: the CYBHi had 63, and the FRGC had over 300 individuals. Considering that and after the preprocessing (filtering) steps, the final number of chimeric individuals was 56. This value was determined by the number of individuals remaining in the base CYBHi dataset after the preprocessing. The samples for each chimeric individual were also limited to the worst case scenario (the individual with the minimum number of samples among all datasets). For our specific case, the number of chimeric individuals was limited by the amount of valid ECG individuals (CYBHi), while the amount of samples per chimeric individual was limited by ECG or face, depending on the combination.

Table 2 shows the results acquired for all modalities’ combinations in three different scenarios. The first column is related to the test conducted on the data used for training (intra-session in the training data), a test over data that were never seen in the training data, referenced as the test (intra-session in the test data), and the last one, where training against test data was used. In the last scenario (inter-session), the test data worked like a probe in a biometric system and the training data as a gallery.

To ensure a statistically fair comparison for each scenario, the experiments were executed 30 times (mean ± standard deviation), which means that each execution used a different combination of the chimeric dataset. We used Student’s *t* to compare the results assuming EER in intra-session and inter-session separately. We compared the lowest EER along with the other results. The best results are highlighted in Table 2. We emphasize that, according to the performed test, there was no statistical significance between the highlighted results in red. In the next sub-sections, we explore all modalities’ combinations.

### 6.1. One Modality

Since the model was overfitted for the training data, it was expected that the intra-session scenario for the same data used to construct the model would lead to better figures. Contrary to this, the inter-session scenario was the closest to the real scenario. For the inter-session scenario, each sample in the test dataset was compared to all samples in the training dataset. Thus, from a biometric system point of view, the test dataset samples played the role of the probe, while the training dataset played the role of the gallery. In the inter-session scenario, the samples were captured at intervals of days or months apart, and therefore, it was more likely to have more intra-class noise, which explains the less favorable results in this scenario.

Analyzing the results for the modalities in an isolated manner, we noticed that the standard deviation was more significant for the ECG modality. We hypothesize that individuals of the type *lamb* and *goat* were more difficult for the ECG dataset than the other datasets.

### 6.2. Two Modalities

Among all presented modality combinations, the fusion of the eye and the face was the most natural/practical. Very often, when a picture of a face is captured, it is intrinsic that the eye is also captured. Both face and eye are well established as strong biometrics. The results presented here (Table 2) support the strength of this fusion, reducing the mean EER acquired with face recognition on FRGC by more than 45% and 90% for the eye.

Even though the most natural fusion is considered the face modality along with the eye, adding the ECG modality to the system can offer significant gains, and it seems to be feasible from the practical point of view. By Table 2, one can see that in addition to reducing the error, the addition of the ECG caused a reduction in the standard deviation and, therefore, a more robust and accurate approach.

### 6.3. Three Modalities

The fusion of ECG with face or eye or both modalities is a feasible fusion since it can be implemented in the real world with accessible mobile devices. The system may be built over a mobile system, where the ECG is captured by off-the-person sensors, such as shown in Figure 1, and the eye/face by the camera of a smartphone.

Robustness and enhancement of recognition are delivered with such a kind of fusion. It also can be used for spoofing detection [14,38]. There are also works that have explored the ECG signal as a manner to detect if a person is alive or not, also known as liveness detection [39].

The ECG signal fusion with eye and face delivers advantages regarding verification rate (in terms of EER). The achieved results showed how two strong biometrics, such as face and eye, still benefited from the fusion with the ECG signal. Since the acquisition of the eye is intrinsic to the acquisition of the face, it is natural to introduce the eye into the fusion of ECG and face. This addition resulted (all three modalities combined) in even better performance, as can be seen in Table 2.

**Figure 9:**
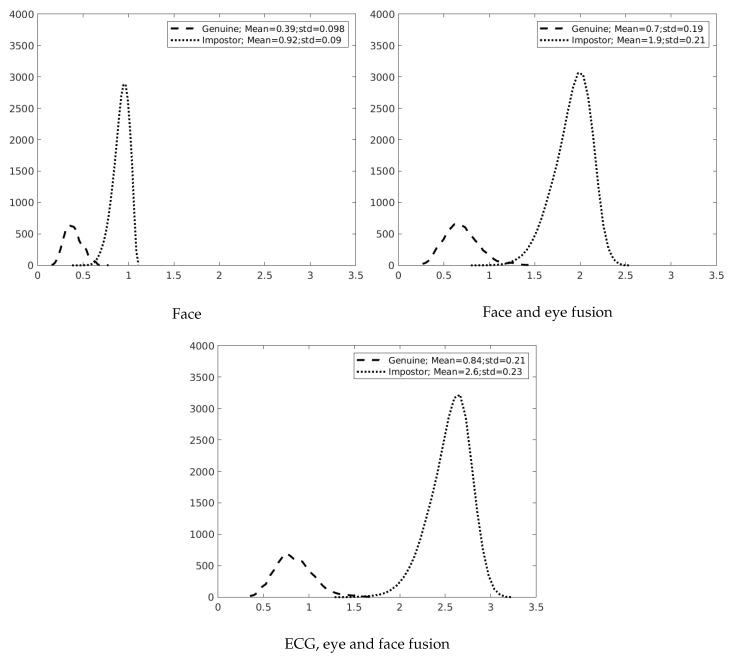
Distribution impostor/genuine curves from the inter-session scenario. The fusion is at the score level using the sum rule.

Figure 9 shows the evolution regarding the addition of the eye and, thereafter, the ECG. It was possible to observe how the distance between the genuine and impostor distribution curves increased when modalities were included. One can see a reduction of the overlap among genuine and impostor distribution curves when the eye modality is combined with face. The overlap between the two curves was even smaller when the ECG signal was included.

In addition to the results, the present study investigated the fusion considering only 56 individuals, which hindered the study regarding scalability. It is important to evaluate the proposed methodology in large chimeric datasets (>1000 individuals).

### 6.4. *Doddington Zoo* Analysis

When datasets are created by using a random procedure, it is not ensured that the dataset is homogeneous, that is individuals may have one modality that weighs more in the result [40]. The *Doddington Zoo* criteria were used aiming at a more homogeneous chimeric dataset, ensuring that during the combination, both modalities were at the same level of difficulty.

It is worth pointing out that this scenario did not necessarily represent the real one; however, it was closer to a worst-case scenario, and it was a more challenging scenario from a biometric perspective. Furthermore, it is important to emphasize that the results on chimeric datasets may diverge from the results on real datasets [41]. In addition, an evaluation protocol should allow a fair comparison between methods and should also facilitate reproducibility. We believe that with the criteria presented in Section 4.3, our protocol favored both reproducibility and a fair comparison among methods.

## 7. Conclusions

In this work, we proposed a simple and reproducible protocol for chimeric datasets’ creation. Moreover, we conducted a statistical evaluation that covered three modalities: ECG signal, eye (periocular region), and face for the biometric system. Since multimodal datasets are scarce in the literature, standardization of protocols is highly desirable.

The chosen modalities allowed the investigation of combining strong and popular modalities (face and eye) along with others less popular ones, such as off-the-person ECG signal. Fusion at two different levels is explored: at the feature level by simple concatenation and at the score level by the sum, min, and multiplication rules. All images and signals were represented by deep learning models extracted using CNN and transfer learning.

Results for the multimodal approach indicated that the fusion of modalities analyzed here was promising. Comparing the multimodal versus unimodal results, the multimodal approach improved the decidability over 28% and 22% over the best unimodal approach for the intra-session of unknown data and inter-session scenario, respectively. Regarding EER, the multimodal approach reduced the error over 11% for the intra-session scenario and by 6% for the inter-session. We emphasize the multimodal approach of ECG, eye, and face, which achieved outstanding results in decidability and EER in both scenarios, intra-session and inter-session. It achieved a decidability of 7.20 ± 0.18, yielding an almost perfect verification system (i.e., EER of 0.20% ± 0.06) in the intra-session scenario for unknown data and a decidability of 7.78 ± 0.78 and an EER of 0.06% ± 0.06 in the inter-session scenario.

The proposed protocol aiming at the creation and evaluation of chimeric datasets forced the creation of homogeneous datasets and therefore allowed a fair comparison between methods. We believe that the protocol favored the reproducibility of the experiments.

Future works include investigating better comprehension of the effects of the chosen fusion and scalability (increase the number of modalities and feature vector size). The scalability can also be explored in the scope of a higher number of individuals (e.g., more than 1000 chimeric individuals). Moreover, another point to study is the use of different distance metrics.

## Figures and Tables

**Table 1:** Comparison of Proença [26] and our approach in the FRGC dataset ran 30 times. The mean and standard deviation are reported.

Method	Decidability	EER
Proença [26]	2.97 ± 0.04	-
Proposed approach	3.62 ± 0.05	5.11% ± 0.23

**Table 2:** Resulting EER and decidability from 30 executions in verification mode of all modalities’ combinations explored by different rules. (con = simple concatenation; min = minimal; mul = multiplication). The best results are highlighted in red. There was no statistical significance among the figures in red; however, they were significantly better than the other figures.

Method	Rule	Intra-Session in Training Data		Intra-Session in Test Data		Inter-Session	
		Decidability	EER (%)	Decidability	EER (%)	Decidability	EER (%)
ECG	-	6.61 ± 0.19	0.94 ± 0.14	5.73 ± 0.21	2.16 ± 0.26	5.70 ± 0.93	1.92 ± 1.23
Eye	-	5.01 ± 0.10	1.43 ± 0.18	3.62 ± 0.12	5.16 ± 0.47	4.32 ± 0.34	3.05 ± 0.84
Face	-	5.46 ± 0.12	0.67 ± 0.12	4.71 ± 0.12	1.57 ± 0.24	5.37 ± 0.57	0.93 ± 0.46
ECG + Eye	min	7.24 ± 0.11	0.46 ± 0.08	6.54 ± 0.20	1.16 ± 0.15	6.35 ± 0.66	1.00 ± 0.49
	mult	6.49 ± 0.10	0.08 ± 0.02	5.97 ± 0.10	0.49 ± 0.10	6.10 ± 0.22	0.28 ± 0.19
	sum	7.62 ± 0.12	0.07 ± 0.02	5.86 ± 0.16	0.58 ± 0.11	6.51 ± 0.48	0.30 ± 0.17
	con	7.55 ± 0.19	0.43 ± 0.08	6.41 ± 0.23	1.34 ± 0.15	6.50 ± 0.97	1.06 ± 0.73
ECG + Face	min	6.89 ± 0.15	0.62 ± 0.10	6.18 ± 0.21	1.41 ± 0.17	6.01 ± 0.75	1.21 ± 0.56
	mult	7.44 ± 0.11	0.08 ± 0.03	6.94 ± 0.11	0.36 ± 0.08	6.97 ± 0.39	0.21 ± 0.14
	sum	8.42 ± 0.18	0.04 ± 0.02	7.30 ± 0.18	0.20 ± 0.06	7.78 ± 0.78	0.10 ± 0.09
	con	7.33 ± 0.21	0.44 ± 0.10	6.31 ± 0.24	1.31 ± 0.16	6.41 ± 1.04	1.06 ± 0.71
Eye + Face	min	5.99 ± 0.10	0.48 ± 0.06	4.38 ± 0.10	2.10 ± 0.22	5.29 ± 0.43	1.10 ± 0.42
	mult	5.86 ± 0.11	0.19 ± 0.04	4.72 ± 0.10	1.76 ± 0.26	5.38 ± 0.27	0.73 ± 0.36
	sum	6.66 ± 0.11	0.18 ± 0.04	4.60 ± 0.12	1.80 ± 0.27	5.81 ± 0.52	0.72 ± 0.35
	con	6.56 ± 0.11	0.17 ± 0.05	4.67 ± 0.11	1.27 ± 0.21	5.93 ± 0.60	0.50 ± 0.29
ECG + Eye + Face	min	7.35 ± 0.11	0.36 ± 0.07	6.63 ± 0.19	1.06 ± 0.12	6.47 ± 0.60	0.82 ± 0.37
	mult	5.63 ± 0.10	0.01 ± 0.00	5.28 ± 0.09	0.18 ± 0.04	5.47 ± 0.13	0.06 ± 0.06
	sum	8.73 ± 0.12	0.01 ± 0.01	6.31 ± 0.15	0.24 ± 0.05	7.56 ± 0.59	0.07 ± 0.06
	con	7.95 ± 0.22	0.26 ± 0.06	6.68 ± 0.24	0.90 ± 0.11	6.92 ± 1.07	0.65 ± 0.44

## Abbreviations

The following abbreviations are used in this manuscript:

CNN	Convolution Neural Network
CYBHi	Check Your Biosignals Here initiative
DET	Detection Error Trade-off
ECG	Electrocardiogram
EER	Equal Error Rate
FAR	False Acceptance Rate
FEyG	Face, Eye, and ECG
FRR	False Rejection Rate
FRGC	Face Recognition Grand Challenge
iBUG	Intelligent Behaviour Understanding Group
MobBIO	Multimodal Database Captured with a Portable Handheld Device
MOBIO	Mobile biometrics
ORL	Olivetti Research Ltd
PCA	Principal Component Analysis
PIN	Personal Identification Number
PRR	Periocular Region Recognition
SOTA	State-Of-The-Art
WVU	West Virginia University Biometrics
